# PD-1 blockade in combination with zoledronic acid to enhance the antitumor efficacy in the breast cancer mouse model

**DOI:** 10.1186/s12885-018-4412-8

**Published:** 2018-06-19

**Authors:** Yuan Li, Yang Du, Ting Sun, Huadan Xue, Zhengyu Jin, Jie Tian

**Affiliations:** 1 Department of Radiology, Peking Union Medical College Hospital, Chinese Academy of Medical Sciences & Peking Union Medical College, No.1 Shuaifuyuan, Dongcheng District, Beijing, 100730 China; 2 Key laboratory of Molecular Imaging of CAS, The State Key Laboratory of Management and Control for Complex Systems, Institute of Automation, Chinese Academy of Sciences, No. 95 ZhongGuanCun East Road, Beijing, 100190 China

**Keywords:** PD-1, Zoledronic acid, Breast cancer, Therapy, Checkpoint inhibitior

## Abstract

**Background:**

Blockade of PD-1 receptor may provide proof of concepts for the activity of an immune-modulation approach for the treatment of breast cancer (BC). Zoledronic acid (ZA) has been proven to inhibit angiogenesis, invasion, and adhesion of tumor cells. The aim of this study was to investigate the potential of monoclonal antibody against T cell checkpoint PD-1 in combination with chemotherapeutic drug ZA in BC mouse model.

**Methods:**

The 4 T1-fLuc mouse BC model was used in this study. The anti-tumor efficacy of anti-PD-1 antibody alone or in combination with ZA was monitored by measuring bioluminescence imaging (BLI) and tumor volume. At the end of study, the flow cytometry was used to determine the immune cell population in tumors after different treatment.

**Results:**

The results showed that mice treated with the combination therapy of anti-PD-1 antibody plus ZA exhibited better antitumor response compared to untreated controls or single therapy with no obvious toxicity.

**Conclusion:**

Our study provides preclinical evidence for the enhanced BC treatment benefit through targeting co-signal molecules by combining anti-PD-1 antibody plus ZA treatment.

## Background

Breast cancer (BC) is the highest incidence of female malignant neoplasia in developed countries and remains the leading causes of cancer death among women in less developed countries. Despite many undeniable therapeutic successes obtained, such as surgery, chemotherapy or radiation therapy, BC still remains one of the major threats to female health [1, 2]. Therefore, it is urgent to find new and potential BC treatment strategies. Immunotherapy is an attractive and promising method for tumor management, which could identify and destroy tumor cells and prevent recurrence and metastatic by exploiting the ability of the immune system. Currently, the blockade of immune checkpoints is the most promising and attractive approach of immunotherapy in oncology [3].

Monoclonal antibodies (mAbs) directed against the programmed cell death protein-1 (PD-1) and against the cytotoxic T lymphocyte-associated antigen-4 (CTLA-4), are exhibiting promising cancer treatment effects, enhance antitumor immunity and improve patient survival, therefore have been approved for the therapies of non-small cell lung cancer and melanoma in clinics [4–6]. PD-1/PD-L1 results in negative regulation of T cells primarily within the tumor microenvironment. The blocking of PD-1 receptor may provide evidence for the activity of immune-modulation in the treatment of BC [7]. In addition, the inhibition of PD-1 signals has shown extremely promising signs of activity in BC [6]. Currently, two mAb treatments for the PD-1 (Pembrolizumab and Nivolumab) are being investigated for clinical use. Pembrolizumab has a significant application prospect in patients with recurrent and/or metastatic head and neck squamous cell carcinoma [8]. Due to its early success, more trials have been conducted to evaluate the efficacy of Nivolumab combined chemotherapy and/or radiation for definitive therapy in locoregionally advanced cancers are currently underway [9]. Allthough these results are encouraging, it remains to be determined in which therapeutic regimen blockade of PD-1 receptor will eventually to improve the prognosis of patients with the greatest impact: as a single agent, or combined with chemotherapy.

Zoledronic acid (ZA) is a third generation drug approved by FDA, typically used in the treatment and prevention of pathologic fractures and osteoporosis [10]. Additionally, it has found that ZA is an anti-resorptive drug that can directly target the tumor, improve immunosurveillance against tumor and to modulate macrophage differentiation microenvironment, target endothelial progenitor cells interfering with their differentiation, thus impair their supportive roles in cancer cells escaping from primary sites [11–13]. ZA has been used to treat many solid tumors, such as lung cancer, prostate cancer, colorectal cancer and recently in clinical trials as an adjuvant therapy for early BC [14, 15]. It was reported that ZA can prevent the tumor promoting effects of mesenchyme stem cells (MSCs) [16].

To further increase the immunotherapeutic efficacy, it was suggested that immunotherapy can be combined with new or standard therapies, with schedule and timing rationally designed. Based on the theories underlying the role of PD-1 and ZA as described above, antibodies blocking PD-1 and ZA are expected to comprise the next generation of therapy against human cancer. In this study, we tested the hypothesis that combined PD-1 antibody and ZA treatment for the treatment of BC.

## Methods

### Materials

Anti PD-1 mAb was obtained from BioXcell (West Lebanon, NH). ZA was got from Sigma Aldrich (St. Louis, MO, USA).

### Cell culture

4 T1-fLuc cells (ATCC® CRL-2539TM) were got from the American Tissue Type Culture Collection (Manassas, VA, USA) and they were stably transfected with firefly luciferase reporter gene. 4 T1-fLuc cells were cultured in RPMI-1640 medium (HyClone, Thermo Scientific, USA) contain 10% foetal calf serum (FCS; HyClone, Thermo Scientific) at 37 °C in a 5% CO_2_ incubator. In a solid tumor model, mice were implanted subcutaneously (s.c.) in the right flank with 1 × 10^6^ Cells in 100 μl PBS.

### Establishment of the BC tumor bearing mouse model

Five-six weeks old female Balb/c mice were obtained from the Department of Experimental Animals, Peking University Health Science Center (Bejing, China). All animal protocols were approved by the Institutional Animal Care and Use Committee in Peking University (Permit Number: 2011–0039), and all procedures were in accordance with approved guidelines. Mice were randomly divided into control and treatment groups.

### Anti-PD-1 mAb and ZA treatment

One day before the injection of tumor cells, mice were treated with ZA in 100 μl phosphate-buffered saline (PBS) intraperitoneally (i.p.) at a dose of 100 μg/kg every 2 days after the tumor inoculation (ZA group, *n* = 10; Anti-PD-1 mAb plus ZA, n = 10). Anti-PD-1 antibody was injected i.p. at a dose of 200 μg/kg after the tumor cell injection (Anti-PD-1 mAb group, n = 10) every 2 days. Control group (n = 10) was injected i.p. with equal volume of PBS.

### Mice body weight and tumor volume measurement after different treatment

Electronic balance was used to measure mice body weight every 3 days. The tumor volume was estmated by measuring the largest (a) and smallest (b) diameters to calculate the tumor volume every 3 days, and the tumor volume calculated according to the following formula: Tumor Volume (mm^3^) = a × b^2^/2.

### Bioluminescence imaging (BLI)

For the in vivo drug treatment evaluation among 4 groups (10 mice each group), Xenogen IVIS Lumina II system (Perkin Elmer, Waltham, MA, USA) was used to monitor BLI as detailed previously [17] every 3 days during drug treatment. D-Luciferin was injected i.p. at a dose of 150 mg/kg and after 10 min mice were imaged dynamically during drug treatment. The imaging signal of regions of interest (ROI) was quantified by the mean photons per second per square centimeter per steradian (p/s/cm^2^/sr).

### Flow cytometry

Tumor-infiltrating lymphocytes (TILs) were isolated from 4 T1 tumors after treatment. Cells were stained with the following antibodies: CD45-e450, CD3-percy525, CD8-percy7, CD25-APCcy7, CD11b-APC (eBioscience, San Diego, CA). The flow cytometry was performed by using FACS Aria III (BD Biosciences, San Jose, CA, USA).

### Histology examination and immunofluorescence staining

After the in vivo experiments, the tumors and also major organs including heart, liver, spleen, lung, and kidney were collected and fixed in the formalin. The paraffin sections were cut at 6–8 μm thickness and stained with hematoxylin and eosin. Microscope (Leica, Wetzlar, Germany) was used to take the images. In additon, tumors were excised, embedded in optimal cutting temperature (OCT) medium, subjected to immunofluorescence staining.

### Serum ELISA analysis of IL-18 and IFN-γ

Levels of plasma interleukin (IL)-18 and interferon (IFN)-γ of mouse origin were analyzed using the Quantikine ELISA kits (R&D Systems). All analyses were in triplicate.

### Statistical analysis

GraphPad Prism V5.0 (GraphPad Software, Inc., San Diego, CA) was used to perform statistical analyses. Results are expressed as the means and standard error of the mean (SEM). One- and two-way analyses of variances (ANOVA) and Tukey’s multiple comparisons test or Student’s t –test were used for data analysis to determine statistical significance between treatment groups. *p* values < 0.05 were considered statistically significant.

## Results

### Combination therapy group showed the most effective antitumor effects monitored using BLI and the tumor volume

**Fig. 1:**
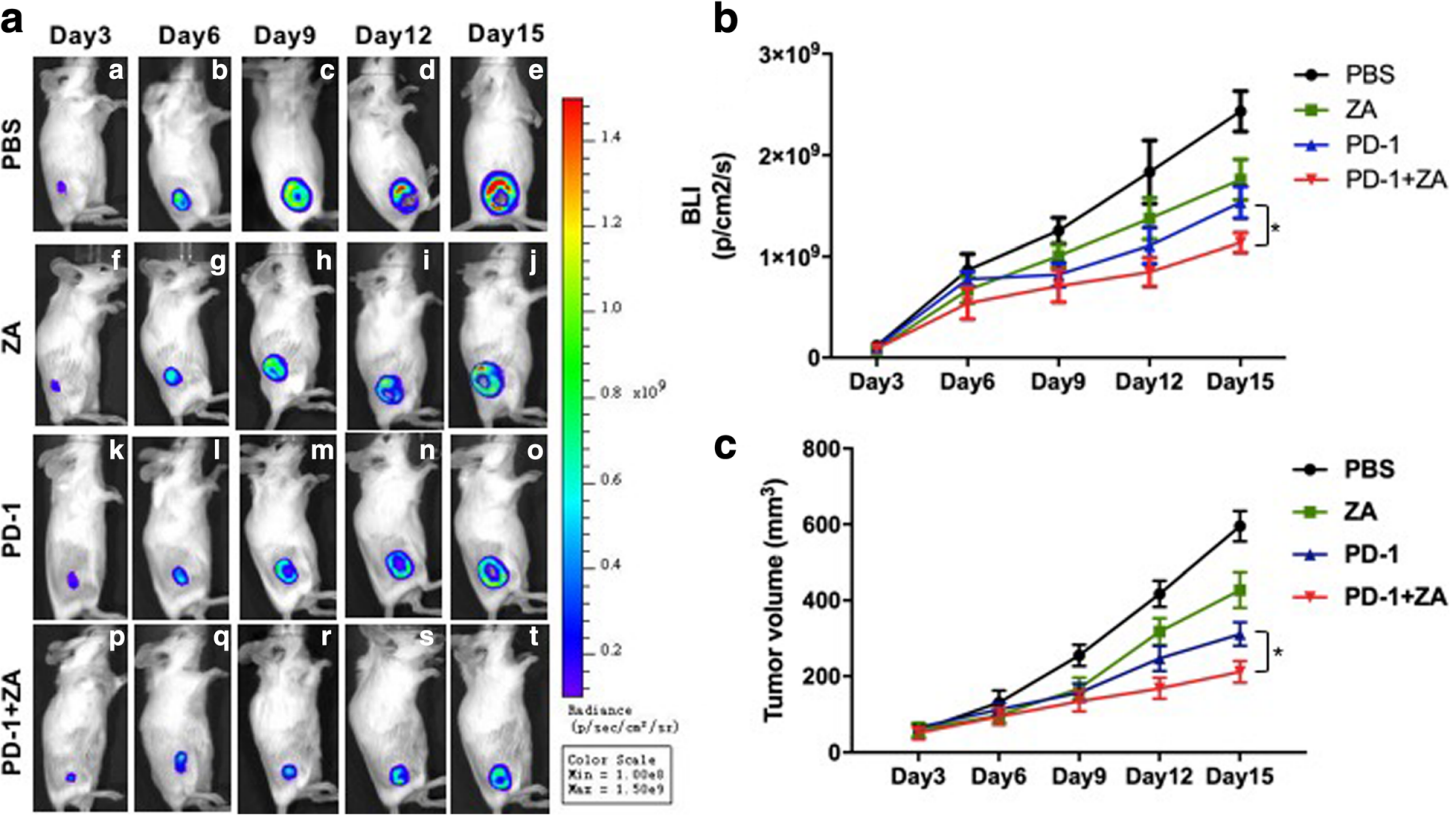
Representative BLI and tumor volume after different treatments for 12 continuous days. **a**. The typical BLI images of each group after different treatments; **b**. Quantification of the BLI signal of tumors after different treatments; **c**. Tumor volume measurement at different time points. The data are presented as the means ± SEM (* *p* < 0.05)

In order to evaluate the different therapeutic efficacy BLI is used which is a sensitive indicator of tumor growth. In the control group, the BLI light intensity greatly increased during the 15-day observation (Fig. 1a (a)–(e)), whereas the BLI light intensities of ZA (Fig. 1a (f)–(j)), the anti-PD-1 mAb (Fig. 1a (k)–(o)) and the anti-PD-1 mAb plus ZA (Fig. 1 (p)–(t)) treatment groups increased slowly compared with the control group during 15 day-treatment. The BLI light intensity of tumors was further calculated and the results showed that anti-PD-1 mAb plus ZA treatment exhibited the lowest light intensity compared with other groups. Compared with the control group and the single treatment groups, the tumor growth inhibition effect was dramatic when the anti-PD-1 mAb were combined with ZA treatment.

The tumor volume was also measured dynamically for the assessment of the anti-tumor activity of anti-PD-1 mAb and ZA. The results were consistent with the in vivo BLI observation, showing that the most effective antitumor effects were the combination of anti-PD-1 mAb and ZA treatment (Fig. 1c). Compared with the anti-PD-1 mAb or ZA blockade only, and combination therapy groups significantly inhibited the tumor growth.

### The body weight of the tumor-bearing mice not be affected after different treatment

**Fig. 2:**
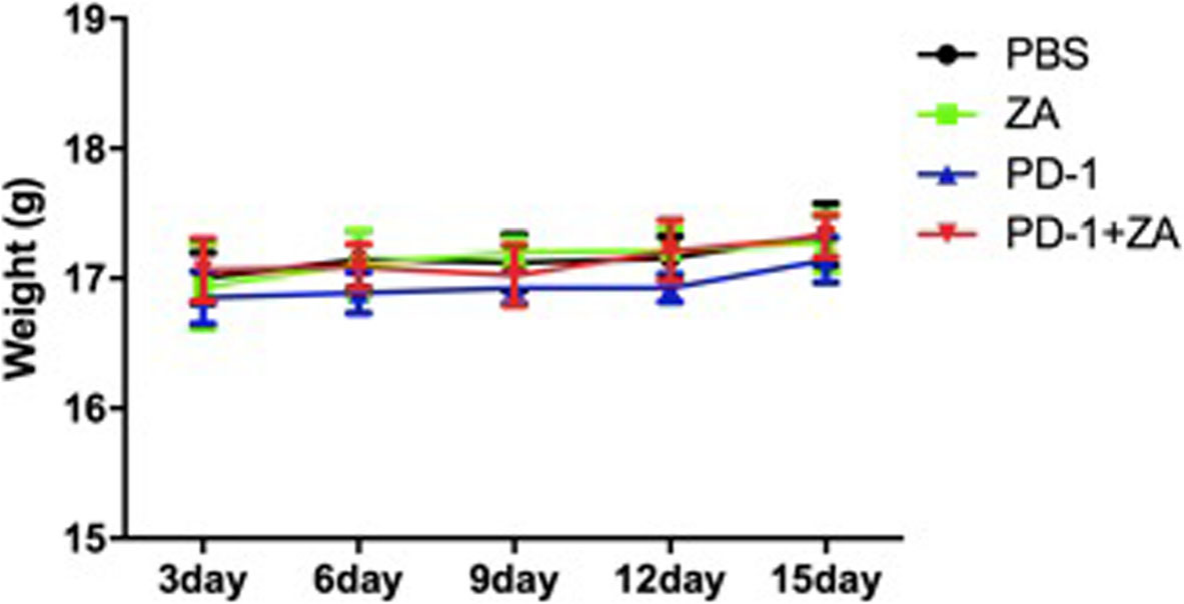
The mouse body weight changes after treatment for 15 continuous days

**Fig. 3:**
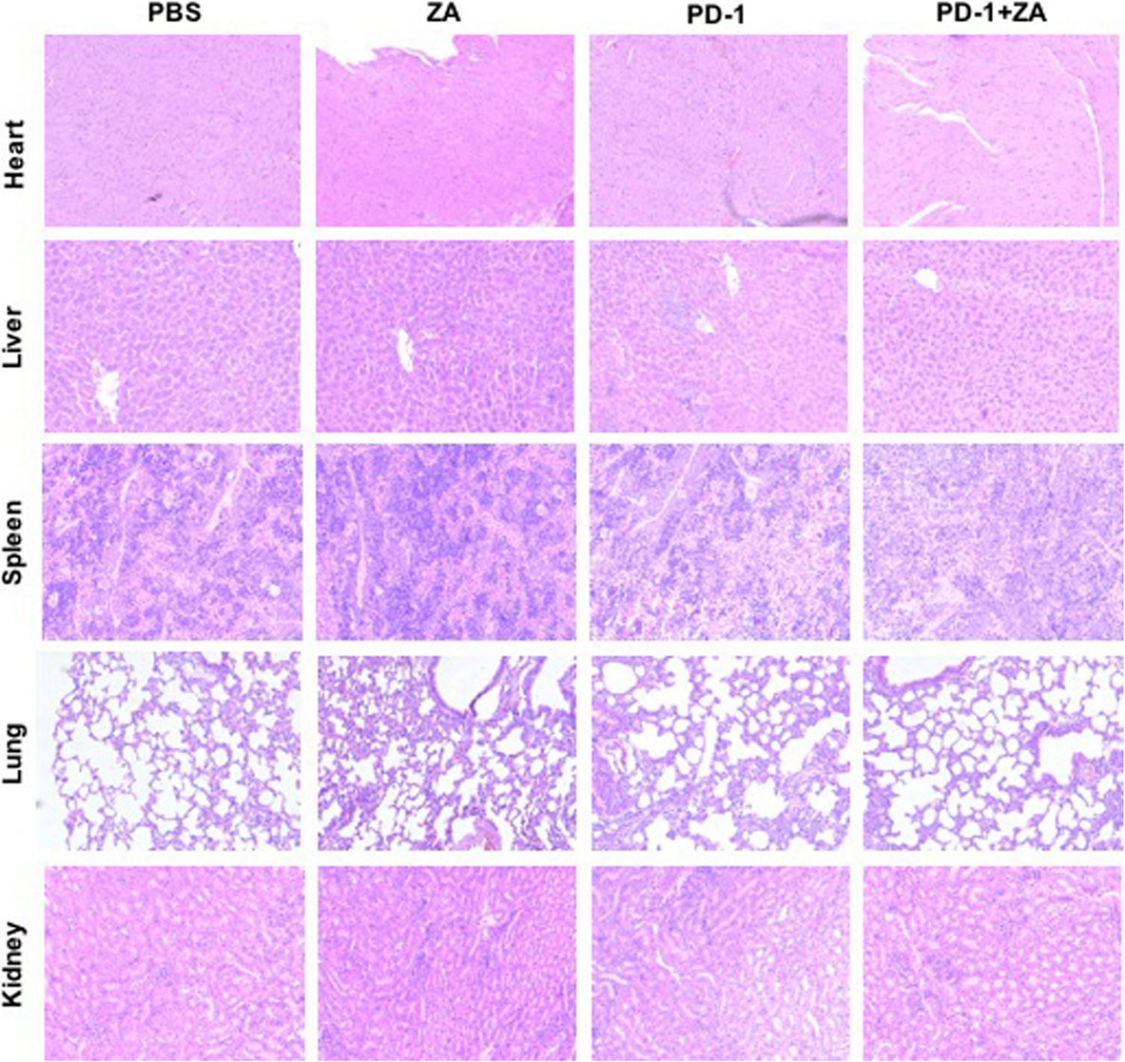
Histological toxicity evaluation of major organs after treatment. H&E staining of the heart, liver, spleen, lung, and kidney harvested from different groups of mice at 15 day after different treatment

Moreover, the safety of treatment was also evaluated dynamically over a 15-day observation period. We found that the behavior and the body weight of the tumor-bearing mice not be affected (Fig. 2). No noticeable tissue damages or any other toxic effects on the major organs (Fig. 3) was founded, which indicate that the dosing regimens were well tolerated with no serious side effects.

### Flow cytometry analysis and immunofluorescence staining analysis of tumor infiltrating lymphocytes (TILs) and macrophages

**Fig. 4:**
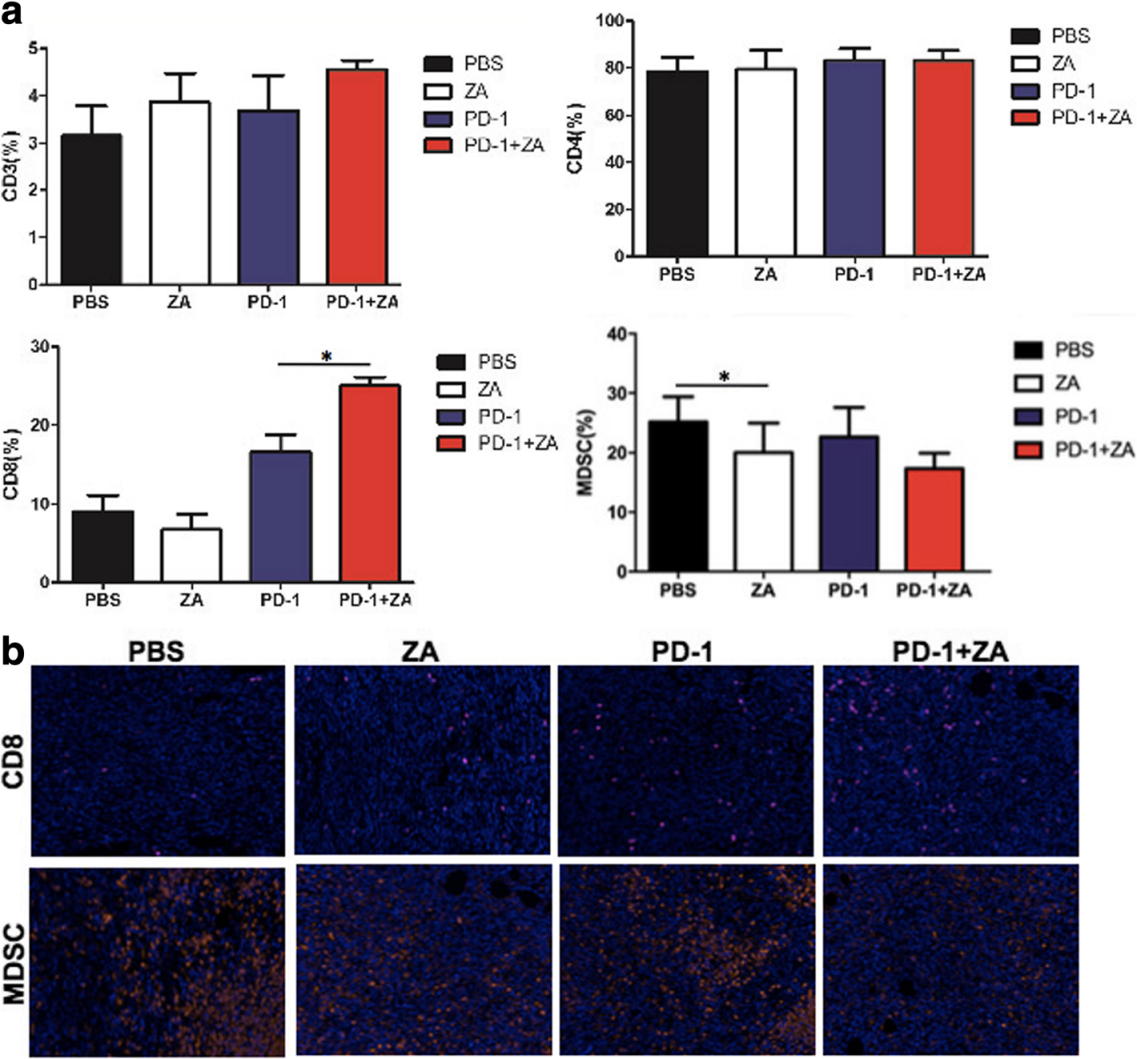
The analysis of TILs in tumors after different treatments using flow cytometry and immunofluorescence staining. **a** Flow cytometry data for the CD3^+^ T cell population, CD8^+^ in the CD3^+^ T cell population, CD4^+^ in CD3^+^ T cells, and MDSC cells. *P < 0.05. **b** Immunofluorescence staining of CD8^+^ and CD11b^+^ MDSC cells in the tumor tissues from 4 T1 mice after different treatments

Moreover, the effects of anti-PD-1 mAb and ZA on TILs using flow cytometry analysis and immunofluorescence staining (Fig. 4). There were relatively more CD8^+^ T cells in the CD3^+^ T cell population in the anti-PD-1 mAb plus ZA group compared to the anti-PD-1 mAb and ZA single treatment groups. There was no difference in terms of CD4^+^ T cell status in the CD3^+^ T cell population. ZA group had a significant decrease in the prevalence of myeloid derived suppressor cells (MDSCs) compared to controls (*p < 0.05) (Fig. 4a). In addition, the immunofluorescence staining result show the increased CD8^+^ T cells and decreased MDSCs in treated mice which further confirm the flow cytometry findings (Fig. 4b), indicating the ability of anti-PD-1 mAb plus ZA to promote CD8^+^ cells infiltration into tumors.

### Increased IFN-r and IL 18 expression in the combination therapy group by serum ELISA analysis

**Fig. 5:**
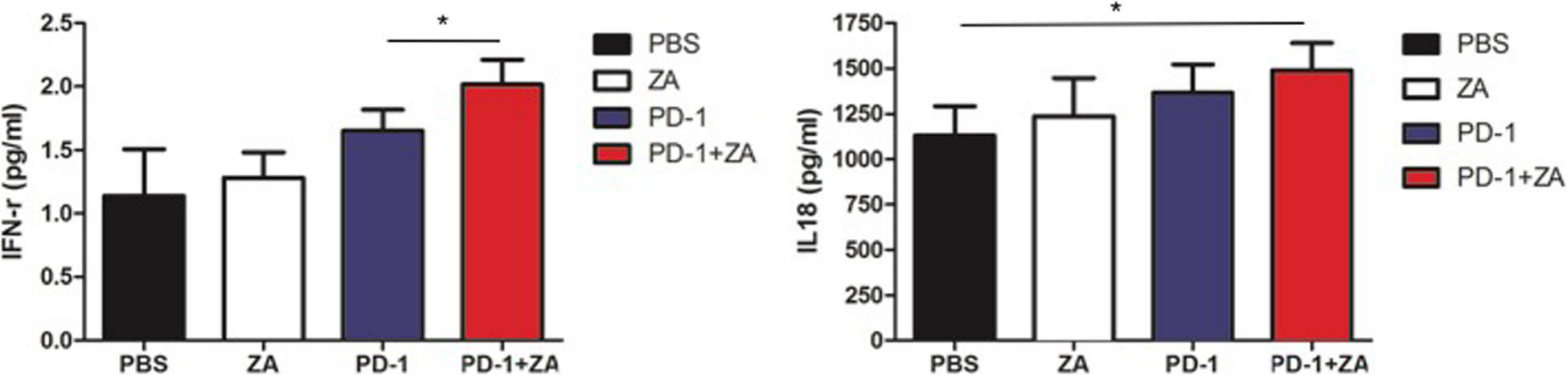
The IFN-γ and IL-18 expression levels in the plasma was examined after different treatments using ELISA, *P < 0.05

Next, ELISA analysis was used to examine the expression levels of interferon (IFN)-γ and Interleukin (IL-18) for a better understanding of how the immune system responds to anti-PD-1 mAb and ZA treatment (Fig. 5). There was approximately 1.7-fold increase in IFN-γ expression in the anti-PD-1 mAb plus ZA-treated mice compared to the control mice and there was significant increase in the anti-PD-1 mAb plus ZA-treated mice compared to the PD-1 treated mice. The result of IL-18 show that there was significant increase in anti-PD-1 mAb plus ZA-treated mice compared to the control treated mice, but there was no significant difference between anti-PD-1 mAb plus ZA-treated mice and the anti-PD-1 mAb treated mice.

## Discussion

Although immune checkpoint inhibitors alone have certain anti-tumor effect and immune modulation function, combination therapy can be more effective, including radiation therapy, biological agents and cell vaccine therapies. This is the first report on the use of anti-PD-1 mAb plus ZA for breast tumor therapy. In this study, we found that mice treated with the combination therapy of anti-PD-1 antibody plus ZA exhibited better antitumor response compared to untreated controls or single therapy as demonstrated by BLI imaging and tumor volume measurement with no obvious toxicity. The possible underlying mechanism was also delineated in this study.

The immune system plays a dual role in tumor immune surveillance and progression, and modulating the immune system is a promising treatment strategy for BC. Immune checkpoint blockade is a new approach for cancer immunotherapy and immune checkpoint blockade expressed on T-cell surface play an important role in this setting sending positive or negative signals to T cells. PD-1and PD-L1/2 axis are negative signals to inhibit T-cell immune response. PD-1/PD-L1 results in negative regulation of T cells primarily within the tumor microenvironment. PD-1, also called CD279, a 55-kDa type I trans-membrane glycoprotein and a member of the immunoglobulin superfamily, has been well characterized as a negative regulator of T cells and functions by delivering inhibitory signals. PD-1 over-expression has been implicated in diverse array of tumor types because of its participation in signaling pathways regulating attenuated CD8^+^ T cell function and enhanced Treg cell activity, which creates an inhibitory environment in the tumors. In a subgroup of thymic T-lymphocytes, PD-1 is produced in a way of constitutive expression, with up-regulated expression found in activated T-cells, B-cells, and myeloid cells [18–20]. These findings suggest that PD-1 is a promising biomarker of BC and a potential therapeutic target itself. On the other hand, anti-PD-1 mAb was reported to show an encouraging antitumor activity in combination with chemotherapy in advanced non-small cell lung cancer [21]. In this study, PD-1 blockade treatment showed significantly delayed tumor growth. IL-18 and IFN-γ levels both play an important role in innate and adaptive immune responses. In the absence of Th1-like cytokines, IL-18 not only accelerates tumor progression but also promote PD-1 express on mature NK cells. As a cytokine, IFN-γ i is critical for natural immunity and adaptive immunity. Once antigen-specific immunity develops, the activated effector T cells could secrete IFN-γ [22]. Serum ELISA analyses of IL-18 and IFN-γ data showed that they were increased in the PD-1 treated mice. These findings suggest that PD-1 is potential therapeutic target itself and indicative of a potential systemic host immune response.

It is found that ZA has direct anti-tumor efficacy, and is often treated as a combination of BC. It was reported that ZA can inhibit the process of multiple intracellular, which was of great significance to cancer cell proliferation and the apoptosis of human leukemic cell lines [23]. In addition, ZA could be a potential anticancer agent by inhibiting angiogenesis, and affects BC metastasis to visceral organs as well as bone through inhibition of migration and invasion of BC cells [24]. Moreover, ZA has been shown to improve immunesurveillance against tumors, to inhibit spontaneous mammary cancerogenesis, opening new possibilities for its treatment application. ZA reduces cancer aggressiveness through abrogating the supportive role of tumor microenvironment. In this study, our flow cytometry data on TILs revealed that there were relatively more CD8^+^ cells, which mainly have an antitumor growth function. The anti-tumor mechanism of PD-1 may be related to the recovery of CD8^+^ cell number, thus relieving the immune suppression and enhancing anti-tumor function. This finding indicated that PD-1 mAb simultaneously works as an adjuvant immunotherapy for ZA chemotherapy. The data suggested that the combination therapy exhibited more efficient antitumor effects with minimal chemotoxicity for the treatment of 4 T1 BC.

## Conclusions

In conclusion, our study indicates that combination therapy with PD-1 blockade and ZA showed significantly inhibit tumor growth, highlighting its promising clinical translational ability for breast tumor management. Our results reveal a potential strategy for immunotherapy with ZA for breast cancer.

## Abbreviations

BC: Breast cancer
BLI: Bioluminescence imaging
CTLA-4: Cytotoxic T lymphocyte-associated antigen-4
IFN-γ: Interferon-γ
IL-18: Interleukin 18
mAbs: Monoclonal antibodies
MSCs: Mesenchyme stem cells
PBS: Phosphate-buffered saline
PD-1: Programmed cell death protein-1
ROI: Regions of interest
RPMI: Roswell Park Memorial Institute
ZA: Zoledronic acid
